# Bacterial Mediated Rapid and Facile Synthesis of Silver Nanoparticles and Their Antimicrobial Efficacy against Pathogenic Microorganisms

**DOI:** 10.3390/ma14102615

**Published:** 2021-05-18

**Authors:** Md. Amdadul Huq, Shahina Akter

**Affiliations:** 1 Department of Food and Nutrition, College of Biotechnology and Natural Resource, Chung-Ang University, Anseong 17546, Korea; 2 Department of Food Science and Biotechnology, Gachon University, Seongnam 461-701, Korea

**Keywords:** *Paenarthrobacter nicotinovorans* MAHUQ-43, extracellular synthesis, AgNPs, antimicrobial activity, *Bacillus cereus*, *Pseudomonas aeruginosa*

## Abstract

In the present study, silver nanoparticles (AgNPs), biosynthesized using culture supernatant of bacterial strain *Paenarthrobacter nicotinovorans* MAHUQ-43, were characterized and their antimicrobial activity was investigated against both Gram-positive *Bacillus cereus* and Gram-negative bacteria *Pseudomonas aeruginosa*. Bacterial-mediated synthesized AgNPs were characterized by UV-Visible (UV-Vis) spectrophotometer, field emission-transmission electron microscopy (FE-TEM), energy dispersive X-ray (EDX), X-ray diffraction (XRD), Fourier transform infrared (FTIR) spectroscopy, and dynamic light scattering (DLS) analysis. The UV-Vis spectral analysis showed the absorption maxima at 466 nm which assured the synthesis of AgNPs. The FE-TEM analysis revealed the spherical shape of nanoparticles with the size range from 13 to 27 nm. The EDX and XRD analysis ensured the crystalline nature of biosynthesized AgNPs. The FTIR analysis revealed the involvement of different biomolecules for the synthesis of AgNPs as reducing and capping agents. The bacterial-mediated synthesized AgNPs inhibited the growth of pathogenic strains *B. cereus* and *P. aeruginosa* and developed a clear zone of inhibition (ZOI). The MIC and MBC for both pathogens were 12.5 µg/mL and 25 µg/mL, respectively. Moreover, field emission scanning electron microscopy analysis revealed that the synthesized AgNPs can destroy the outer membrane and alter the cell morphology of treated pathogens, leading to the death of cells. This study concludes the eco-friendly, facile and rapid synthesis of AgNPs using *P. nicotinovorans* MAHUQ-43 and synthesized AgNPs showed excellent antimicrobial activity against both Gram-positive and Gram-negative pathogens.

## 1. Introduction

Nanotechnology is a rapidly growing field of science that contributed to the synthesis of a wide variety of metal nanoparticles (MNPs). MNPs (1–100 nm) have gained significant interest by researchers due to their various applications in different fields of science. There are several kinds of MNPs including gold, zinc, silver, copper, iron, etc. with unique characteristics [1,2,3,4,5]. Most of these MNPs have various applications in biomedical fields as antioxidant, anticancer, antibacterial, antifungal, tumor detection, drug delivery, biodegradation, wastewater treatment, etc. [6,7,8,9,10]. Among different kinds of MNPs, silver nanoparticles (AgNPs) are widely used nanoparticles (NPs) in different fields of biomedical science as anti-cancer, anti-inflammatory, anti-microbial, wound dressings, drug delivery, biosensors, and catalysis, etc. [11,12,13,14,15,16,17,18]. The truth of these matters has been found in recent studies, where biogenic AgNPs showed strong antimicrobial, antioxidant, and anti-cancer activities [11,12,13]. Moreover, the biogenic AgNPs were effective for the biodegradation of toxic chemicals [14,15]. The biogenic AgNPs also have various applications in different fields of biotechnology such as food preservation, water filtration, sanitization, clothing, cosmetics, nanoinsecticides, nanopesticides, etc. [7,17,19]. Biogenic AgNPs are well-known for their antibacterial activity against both Gram-positive and Gram-negative pathogenic bacteria [2,6,20,21]. The AgNPs have been also reported as potential antibacterial agent against multidrug-resistant bacteria like *Salmonella Typhimurium*, *Staphylococcus aureus, Escherichia coli, Vibrio parahaemolyticus, Enterobacter hormaechei, Klebsiella pneumoniae* etc. [1,2,6,22,23].

The commonly used methods for the synthesis of MNPs are physical and chemical synthesis, employing reagents and extreme pressure or temperature whose function is to reduce the metal ions and stabilize the nanoparticles. During the synthesis process, various hazardous byproducts are also generated [2,3,24,25]. These reagents and byproducts are toxic for human health and the environment, which has led to increasing interest in biosynthesis methods. The biosynthesized nanoparticles have higher stability, lower toxicity with better physicochemical characteristics [26,27,28]. Currently, researchers are focusing more on biological approaches that include plants and their various parts and microorganisms such as bacteria, fungi, algae, etc. for eco-friendly, non-toxic, and facile synthesis of MNPs [26,27,28]. Among the different biological approaches, bacterial-mediated MNPs synthesis is mostly preferred because of their high growth rate, large-scale production, ease of handling, and the possibility of genetic engineering [6,29]. Studies have reported about the synthesis of AgNPs using numerous bacteria including *Paenibacillus anseongensis*, *Novosphingobium* sp., *Microvirga rosea, Pseudomonas* sp., *Sporosarcina koreensis* DC4, etc. [30,31,32,33,34]. Most of these reported studies did not investigate the optimal parameters for the rapid and stable synthesis of nanoparticles. Also, only some of them investigated the antimicrobial activity of synthesized nanoparticles at the screening level, instead of the mechanism level [30,31,32,33,34].

The infectious diseases caused by different bacteria are a serious threat for public health worldwide due to the emergence of multidrug-resistant microorganisms. New therapies are urgently needed to control these multidrug-resistant microorganisms. Biosynthesized AgNPs could be used as a potential antimicrobial agent against such pathogens. In this study, novel AgNPs were biosynthesized using the culture supernatant of strain *P. nicotinovorans* MAHUQ-43 and characterized by different techniques including UV-Vis, FE-TEM, EDX, XRD, FT-IR, and DLS. Antimicrobial efficacy of biogenic AgNPs was investigated against Gram-positive pathogen *B. cereus* and Gram-negative pathogen *P. aeruginosa*. This is the first report for the synthesis of novel AgNPs using *P. nicotinovorans* MAHUQ-43 and their antimicrobial efficacy against pathogenic microorganisms.

## 2. Materials and Methods

### 2.1. Materials

Silver nitrate (AgNO_3_) was bought from Sigma Aldrich (St. Louis, MO, USA). The bacterial culture media such as R2A agar and R2A broth were purchased from MB-cell, (Seoul) South Korea, Mueller-Hinton agar (MHA), Mueller-Hinton broth (MHB), and standard antibiotics were bought from Oxoid Ltd., Basingstoke, England. The pathogenic strains *P. aeruginosa* (ATCC 10145) and *B. cereus* (ATCC 10876) were obtained from American Type Culture Collection (ATCC) (University Boulevard, Manassas, VA, USA).

### 2.2. Isolation and Identification of AgNPs Producing Bacterial Strain MAHUQ-43

The bacterial strains were isolated from soil sample of a maize field, located in Anseong, South Korea using the serial dilution technique. For screening of extracellular synthesis of AgNPs, all isolated strains were cultured separately in 5 mL R2A broth media for 48 h at 30 °C. Then, the culture supernatant was collected and AgNO_3_ solution (1 mM final concentration) was added and incubated in a shaking incubator for 48 h at 30 °C. On the basis of the reduction efficacy of AgNO_3_ to AgNPs, strain MAHUQ-43 was selected as a strong candidate and identified through 16S rRNA gene sequence analysis using bacterial universal primers 27F and 1492R [35]. The 16S rRNA gene sequence of strain MAHUQ-43 was submitted to GeneBank, NCBI. The 16S rRNA gene sequences of related taxa were collected and compared from EzBioCloud server [36]. The phylogenetic tree was constructed by MEGA6 program [37] with the neighbor joining method, to represent the phylogenetic position of strain MAHUQ-43 [37].

### 2.3. Biosynthesis of AgNPs Using Strain MAHUQ-43

For bacteriogenic synthesis of AgNPs, the isolated strain *P. nicotinovorans* MAHUQ-43 was cultured in R2A broth medium (100 mL) in a 250 mL flask for 48 h at 30 °C with 180 rpm. After 48 h (Optical density was approximately 1.0 at 600 nm), the culture supernatant was collected by centrifugation at 9000 rpm for 10 min. Then, freshly made AgNO_3_ solution was added into 100 mL of culture supernatant and the final concentration was adjusted to 1 mM. Subsequently, the reaction vessel (250 mL conical flask) was incubated at 30 °C for 24 h for the complete formation of AgNPs. Initially, the synthesis process was monitored by visual observation through the alteration of color. Finally, the synthesized AgNPs were collected by high-speed centrifugation (13,500 rpm) for 25 min at 10 °C. The collected AgNPs were washed several times using distilled water and then, dried at room temperature to obtain the powder form and finally, used for characterization as well as antimicrobial studies.

### 2.4. Characterization of Synthesized AgNPs

The biosynthesis of novel AgNPs was confirmed using a UV-Vis spectrophotometer (Optizen POP, Mecasys, Daejeon, South Korea) in the range of 300–800 nm. The size and shape of AgNPs were determined by FE-TEM (field emission–transmission electron microscopy) (JEM-2100F, JEOL, Tokyo, Japan), operating at 200 kV. For FE-TEM analysis, AgNPs were dissolved in deionized water and a drop was placed on carbon-coated copper grids and then, dried at room temperature and finally, used to take the TEM images [31]. The EDX (energy dispersive X-ray) spectrum and SAED (selected area diffraction) were performed to examine the metallic nature, elemental compositions, and purity of synthesized AgNPs using the detector attached with FE-TEM. To determine the crystalline nature of biosynthesized AgNPs, XRD (X-ray diffraction) analysis was performed using Cu-Kα radiation generated at 40 kV with 40 mA over the range of 20–80° (2delta) [31,38]. FTIR (Fourier transform-infrared) spectroscopy was used in the range of 4000 to 500 cm^−1^ to find out the functional groups associated with biosynthesized AgNPs [34,39]. Hydrodynamic diameters and polydispersity index (PDI) were investigated through dynamic light scattering (DLS) analysis according to the previous description [31].

### 2.5. Antibacterial Activity of Synthesized AgNPs

Antibacterial activity of the biosynthesized AgNPs was evaluated by disc diffusion method according to the previous description with slight modifications [34]. In brief, the pure colony of *P. aeruginosa* (ATCC 10145) and *B. cereus* (ATCC 10876) was grown in MHB medium for overnight at 37 °C (160 rpm). Then, 100 μL of each culture was spread on the MHA plates and sterile paper discs were placed on the media. Subsequently, 30 μL and 60 μL biosynthesized AgNPs solution (1 mg/mL, dissolved in distilled water) were poured on the paper discs. At the same time, three different standard antibiotics (penicillin G, 10 μg/disc; lincomycin, 15 μg/disc, and vancomycin, 30 μg/disc) were used as controls against both pathogens *P. aeruginosa* and *B. cereus*. All plates were incubated at 37 °C for 24 h to check the zone of inhibition (ZOI). After incubation period, the ZOI was calculated and recorded in millimeters. All the data presented in the current study are the means of three independent replicates.

### 2.6. Determination of Minimum Inhibitory (MIC) and Minimum Bactericidal Concentrations (MBC)

The MIC of synthesized AgNPs against *P. aeruginosa* and *B. cereus* was examined by the broth microdilution assay using 96-well plates [40]. Briefly, both pathogens were grown overnight in MHB medium at 37 °C and then, turbidity was fixed approximately 1 × 10^6^ CFU/mL. About 100 μL of each pathogen (1 × 10^6^ CFU/mL) was added into 96-well plates and an equal volume of synthesized AgNPs solution with different concentrations in the range of 1.56 to 100 μg/mL was added. For control, only medium was used instead of AgNPs solution. Finally, the culture loaded 96-well plate was incubated at 37 °C (160 rpm) for 24 h. Using ELISA plate reader (LabTech 4000) (BMG LABTECH, Ortenberg, Germany), the absorbance (at 600 nm) was taken every 3 h of interval. The lowest concentration of synthesized AgNPs which fully inhibited the growth of the tested pathogens was determined as MIC. The MBC of synthesized AgNPs was calculated by streaking 10 μL of each suspension taken from MIC wells and incubating for 24 h at 37 °C. After incubation period, the minimum concentration of biosynthesized AgNPs that blocked bacterial growth on MHA plates was recorded as the MBC.

### 2.7. FE-SEM Analysis

The structural and morphological changes of AgNPs-treated *P. aeruginosa* and *B. cereus* were investigated by FE-SEM analysis according to the previous description with little modification [40]. Briefly, log-phase cells (approximately 1 × 10^7^ CFU/mL) were treated with bacteriogenic AgNPs at MBC concentration for 6 h at 37 °C. At the same time, untreated cells of both pathogens were maintained as control. The cells were washed several times using PBS (pH 7.0) and fixed using 2.5% glutaraldehyde for 4 h at room temperature. After fixation, the cells were washed several times using PB buffer and subsequently dehydrated with ethanol (30, 50, 70, 80, 90, and 100%) for 10 min each at room temperature. Finally, the dehydrated cells were dried by a desiccator and the samples were coated with gold for morphological and structural observation by FE-SEM (JSM-7100F, JEOL, Japan) [40].

## 3. Results and Discussion

### 3.1. Isolation and Identification of Strain MAHUQ-43

**Figure 1:**
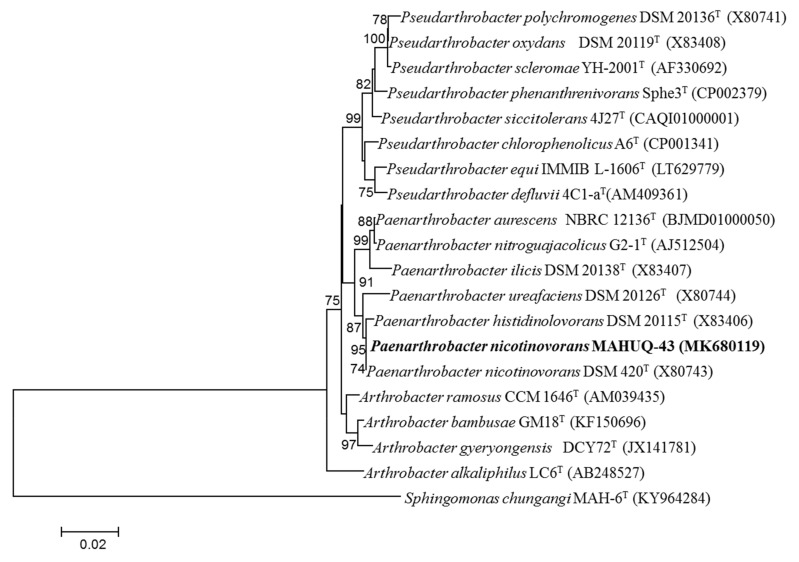
The neighbor-joining (NJ) tree based on 16S rRNA gene sequence analysis showing phylogenetic relationships of strain MAHUQ-43^T^ and the related type strains. Bootstrap values more than 70% based on 1000 replications are shown at branching points. Scale bar, 0.02 substitutions per nucleotide position.

Bacterial strain MAHUQ-43 was isolated from the soil sample of a maize garden and selected for the biologically rapid synthesis of AgNPs. The analysis of nearly complete sequence (1421 bp) of 16S rRNA gene of strain MAHUQ-43 revealed that the isolated strain MAHUQ-43 had maximum similarity with *P. nicotinovorans* DSM 420^T^ (100% similarity) and thereafter referred to as *P. nicotinovorans* MAHUQ-43. The 16S rRNA gene sequence of strain MAHUQ-43 was submitted to the GeneBanK (NCBI) with an accession number MK680119. Phylogenetic analysis using neighbor joining method showed that the strain MAHUQ-43 made cluster with genus *Paenarthrobacter* (Figure 1). Strain MAHUQ-43 was deposited to the Korean Agricultural Culture Collection (KACC 21240).

### 3.2. Bacteriogenic Synthesis of AgNPs

**Figure 2:**
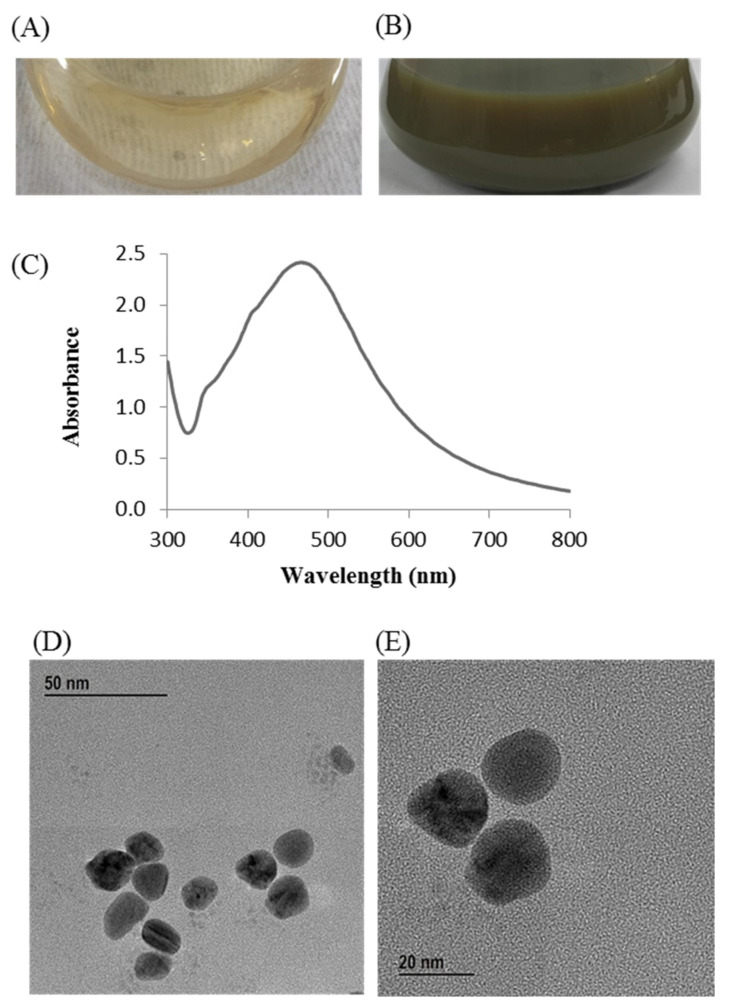
R2A broth with AgNO_3_ as control (**A**), *P. nicotinovorans* MAHUQ-43-mediated synthesized AgNPs (**B**), UV–vis spectra (**C**), and FE-TEM images of synthesized AgNPs (**D**,**E**).

Change in the reaction mixture color during incubation was the primary indication for the biosynthesis of AgNPs. The reaction mixture color turned to dark brown from watery yellow within 24 h of incubation (Figure 2B). On the other hand, no color change was found in the control flask (R2A broth with AgNO_3_) under similar experimental conditions, suggesting no formation of AgNPs (Figure 2A). The synthesized AgNPs were collected by high-speed centrifugation (13,500 rpm for 25 min at 10 °C). Subsequently, the collected nanoparticles were washed several times using distilled water and finally air dried to get powder form for characterizations. The biosynthesis of MNPs using bacteria can be acquired by either extracellular or intracellular processes. Intracellular process of nanoparticles synthesis requires additional downstream steps for the isolation and purification of synthesized nanoparticles that make it complex and costly [41]. On the other hand, extracellular process is a one-step process and do not need any additional downstream steps that make the process facile, rapid, and inexpensive [41]. In this study, extracellular process was used for rapid, facile, and inexpensive synthesis of AgNPs. *P. nicotinovorans* MAHUQ-43 culture supernatant-mediated bacteriogenic synthesis of AgNPs was completed within 24 h which was comparatively a rapid synthesis process than some other microbial-mediated synthesis [30,31,33]. The optimum temperature (30 °C) and AgNO_3_ concentration (1 mM final concentration) for rapid and stable synthesis of AgNPs were examined on the basis of UV-vis spectral analysis (Supplementary Figures S1 and S2). The accurate mechanism behind the synthesis of AgNPs using bacterial culture supernatant is poorly understood. However, previous reports suggest that different extracellular biomolecules including amino acids, proteins, enzymes, and carbohydrates secreted by microorganisms in their culture supernatant are associated with the reduction of Ag+ ions to AgNPs and the stabilization of AgNPs by capping [34,42].

### 3.3. Characterization of Synthesized AgNPs

**Figure 3:**
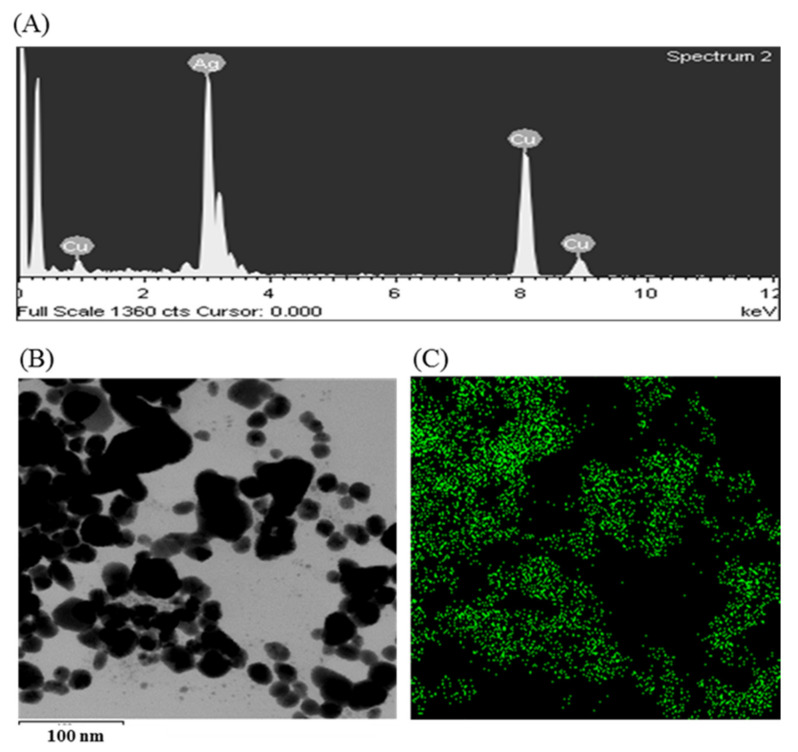
EDX spectrum *P. nicotinovorans* MAHUQ-43-mediated synthesized AgNPs (**A**), TEM image used for mapping (**B**) and distribution of silver in elemental mapping (**C**).

**Figure 4:**
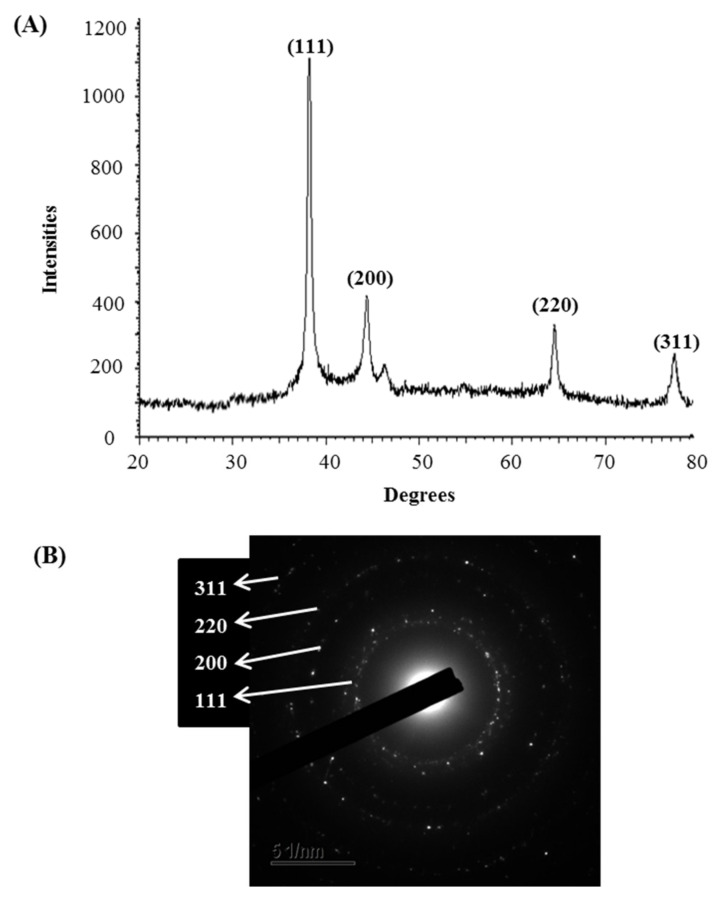
X-ray diffraction pattern (**A**) and SAED pattern (**B**) of *P. nicotinovorans* MAHUQ-43-mediated synthesized AgNPs.

The synthesis of AgNPs was initially confirmed by the development of dark brown color in the reaction mixture (Figure 2B), which occurs due to surface plasmon resonance (SPR), a general feature of AgNPs [43]. The synthesis of AgNPs was further confirmed by UV-Vis spectrophotometer. A strong peak in UV-Vis absorbance was found at 466 nm which confirmed the formation of AgNPs (Figure 2C). Absorption of light on the surface of nanoparticles by the band of electrons vibrating at characteristic modes is known as the surface plasmon resonance (SPR) phenomenon [44]. Similar results were found in the previous studies [31,34]. FE-TEM analysis identified the morphology of synthesized AgNPs, such as shape and size. The TEM images of synthesized AgNPs revealed the spherical shape of nanoparticles and the size was in a range of nearly 13–27 nm (Figure 2D,E). EDX analysis was also carried out to investigate the metallic nature of the biosynthesized nanoparticles. EDX spectrum showed a strong signal at 3 keV, indicating the synthesis of AgNPs (Figure 3A). Some other peaks were also observed in this EDX spectrum due to the use of copper grids (Figure 3A). The elemental mapping results revealed the highest distribution of silver elements in the sample that assured the purity of the sample (Figure 3B,C, Table 1). In the XRD spectrum, four distinct peaks were found at 2θ values of 38.13, 44.76, 64.83, and 77.95º corresponding to the 111, 200, 220, and 311 Bragg’s reflection (Figure 4A) which confirmed the crystalline structure of synthesized AgNPs. The SAED pattern also confirmed the crystalline nature of synthesized AgNP. SAED pattern revealed sharp rings which indicate the crystalline nature of AgNPs and correspond to the reflections of 111, 200, 220, and 311. Similar data were found in the previous reports [31,34].

**Figure 5:**
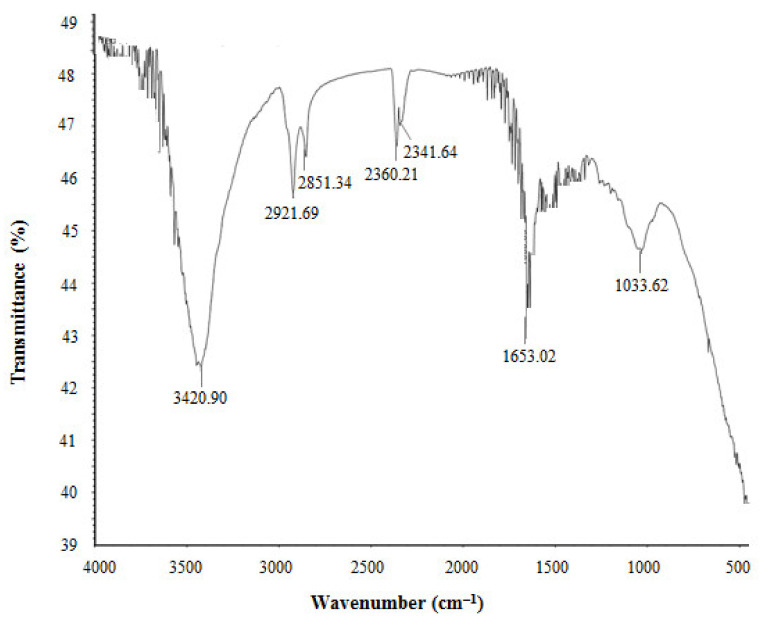
FT-IR spectra of *P. nicotinovorans* MAHUQ-43-mediated synthesized AgNPs.

**Figure 6:**
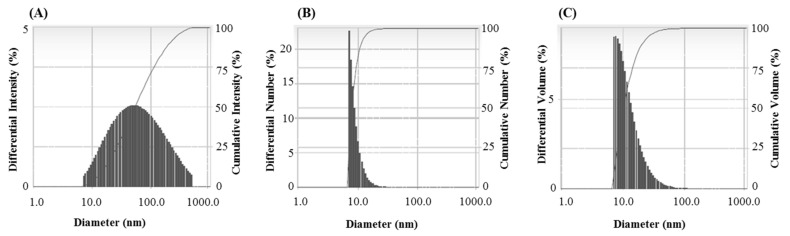
Particles size distribution of *P. nicotinovorans* MAHUQ-43-mediated synthesized AgNPs according to intensity (**A**), number (**B**) and volume (**C**).

The FTIR results revealed different functional groups associated with synthesized AgNPs. FTIR spectrum showed strong vibrational stretches at 3420.90, 2921.69, 2851.34, 2360.21, 2341.64, 1653.02, and 1033.62 cm^−1^ (Figure 5) due to the association of different functional groups. The different absorbance peaks like 3420.90 cm^−1^ for O–H (alcohol) and/or N–H (amine), 2921.69 and 2851.34 cm^−1^ for C–H (alkane), 2360.21 and 2341.64 cm^−1^ for O=C=O (carbonyl bond group), 1653.02 cm^−1^ for N–H (amine), and 1033.62 cm^−1^ for C–O (alcohol/ether) group. These functional groups which are produced by the bacteria in the form of proteins, enzymes, or other metabolites may associate with the reduction and stabilization process of nanoparticles synthesis. Previous studies have shown the similar FTIR spectrums of AgNPs synthesized using microorganisms [31,34]. The particle size of biosynthesized AgNPs was measured by DLS analysis. Figure 6 showed the particle size distribution of bacteriogenic AgNPs through DLS analysis on the basis of intensity, number, and volume. The average particle size of AgNPs was 44.2 nm with polydispersity value (0.443). Similar results were found in previous reports [31,34].

### 3.4. Antibacterial Activity of AgNPs

**Figure 7:**
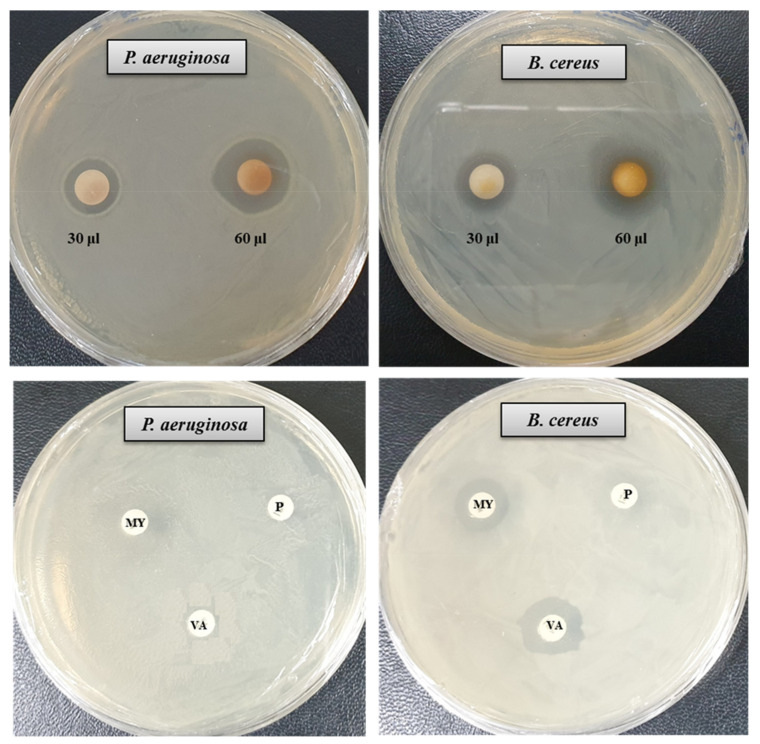
Zones of inhibition of *P. nicotinovorans* MAHUQ-43-mediated synthesized AgNPs (30 μL and 60 μL at 1000 ppm concentrations in water) and commercial antibiotics against *P. aeruginosa* and *B. cereus.* Abbreviation: P (penicillin, G 10 μg/disc), MY (lincomycin, 15 μg/disc), and VA (vancomycin, 30 μg/disc).

The antibacterial activity of bacteriogenic AgNPs was investigated by the disk diffusion method. The potent antimicrobial properties of synthesized AgNPs against *P. aeruginosa* and *B. cereus* were confirmed by the appearance of clear ZOI. A clear ZOI was observed around the paper disks containing AgNPs on MHA plates against pathogenic *P. aeruginosa* and *B. cereus* as shown in Figure 7. The presence of clear ZOI confirmed the complete growth inhibition of *P. aeruginosa* and *B. cereus*. The diameters of ZOI of AgNPs were calculated in millimeters and shown in Table 2. The diameters of ZOI of AgNPs against *P. aeruginosa* and *B. cereus* were 24.7 ± 0.9 and 19.3 ± 1.0, respectively. As control, three standard antibiotics such as penicillin G, vancomycin, and lincomycin were used to check their antibacterial activities against *P. aeruginosa* and *B. cereus.* It was found that all of these three antibiotics did not show any activity against *P. aeruginosa*. In case of *B. cereus*, vancomycin and lincomycin showed week activity but penicillin G did not show any activity (Figure 7, Table 2). The inhibition potential of the synthesized AgNPs against *P. aeruginosa* and *B. cereus* was significantly high compared to that of the tested antibiotics. Findings of this study suggest that the *P. nicotinovorans* MAHUQ-43-mediated synthesized AgNPs were able to control pathogenic *P. aeruginosa* and *B. cereus*.

### 3.5. Determination of Minimum Inhibitory (MIC) and Minimum Bactericidal Concentrations (MBC)

**Figure 8:**
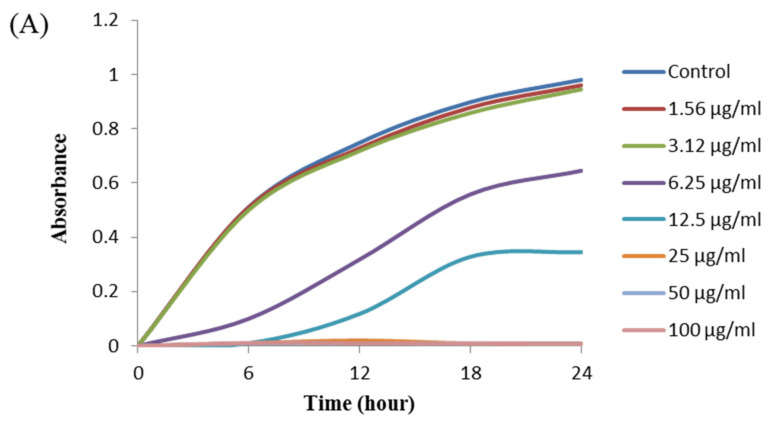

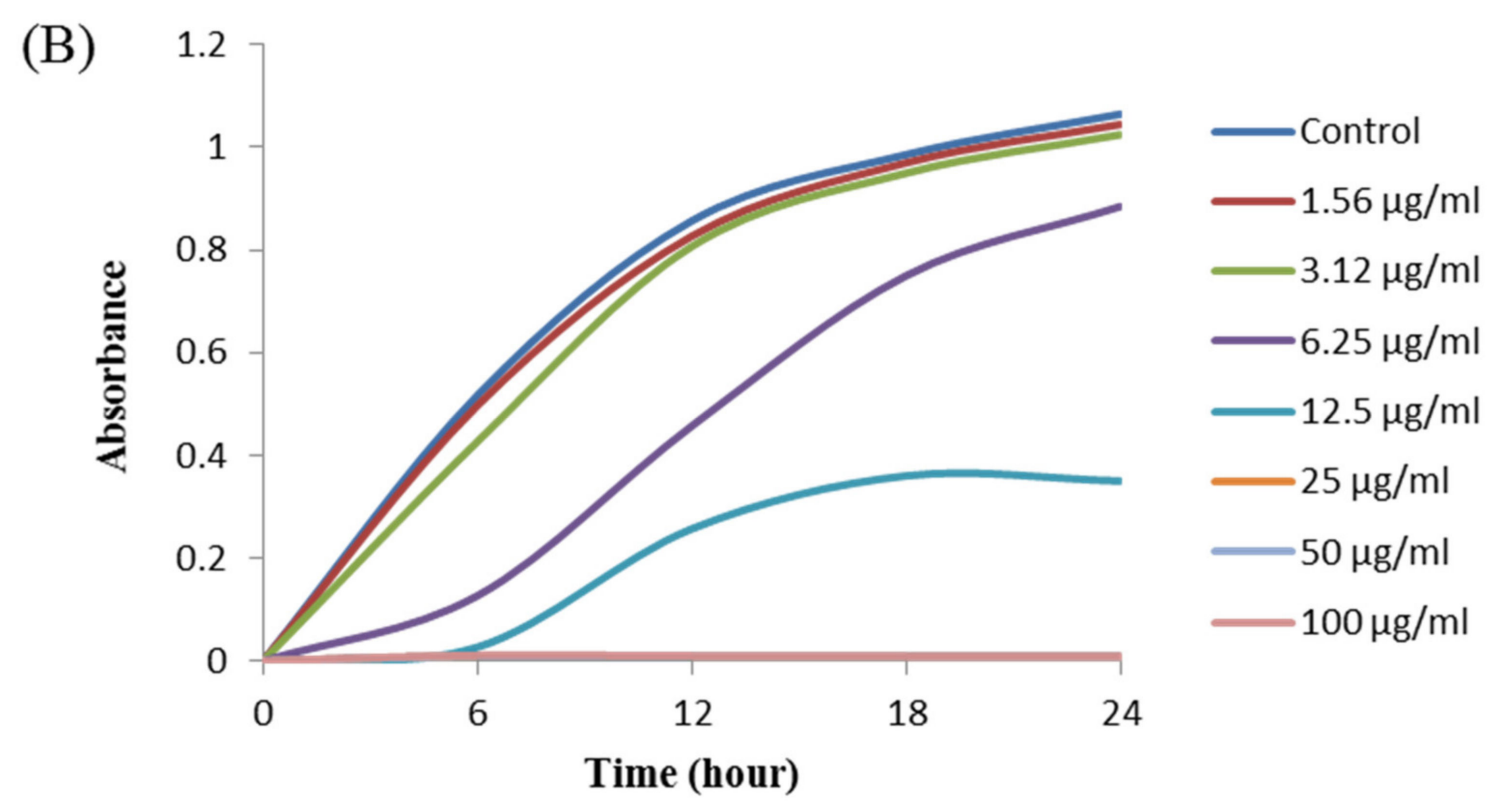
Growth curves of *P. aeruginosa* (**A**) and *B.cereus* (**B**) cultured in MHB with different concentrations of *P. nicotinovorans* MAHUQ-43-mediated synthesized AgNPs to determine MIC.

**Figure 9:**
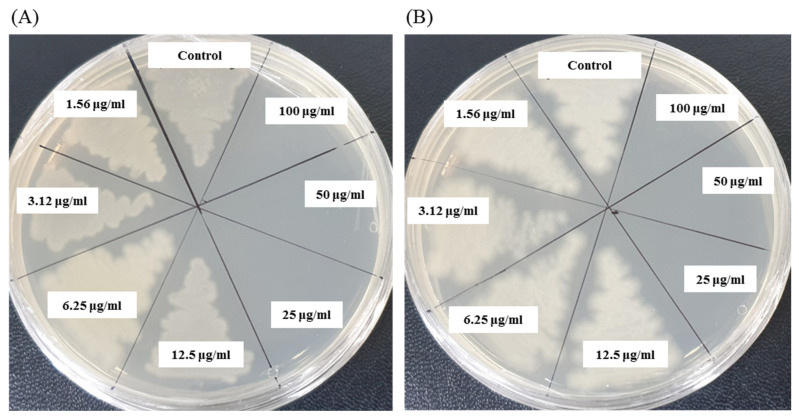
MBC of *P. nicotinovorans* MAHUQ-43-mediated synthesized AgNPs against *P. aeruginosa* (**A**) and *B. cereus* (**B**).

The antibacterial activity of biosynthesized AgNPs was also evaluated by MIC and MBC assay. The MIC is the lowest concentration that inhibits the growth of pathogenic microorganisms. The MIC of synthesized AgNPs for both *P. aeruginosa and B. cereus* was 12.5 μg/mL (Figure 8). This MIC value was significantly lower than some other antimicrobial agents including biosynthesized nanoparticles against both *P. aeruginosa and B. cereus* [45,46,47]. For example, MIC values of biosynthesized ZnO NP were 1600–3200 μg/mL against different strains of *P. aeruginosa* [45]. On the other hand, MIC value of chitosan-based nanoparticles was 125 μg/mL against *B. cereus* [47]. The MBC is the lowest concentration that killed the test microorganisms resulting in no growth on the agar plates. The MBC of synthesized AgNPs was determined as 25 μg/mL for both *P. aeruginosa* and *B. cereus* (Figure 9). This MBC value was also significantly lower than some other antimicrobial agents including synthesized nanoparticles against both *P. aeruginosa and B. cereus* [45,46,47].

### 3.6. FE-SEM Analysis

**Figure 10:**
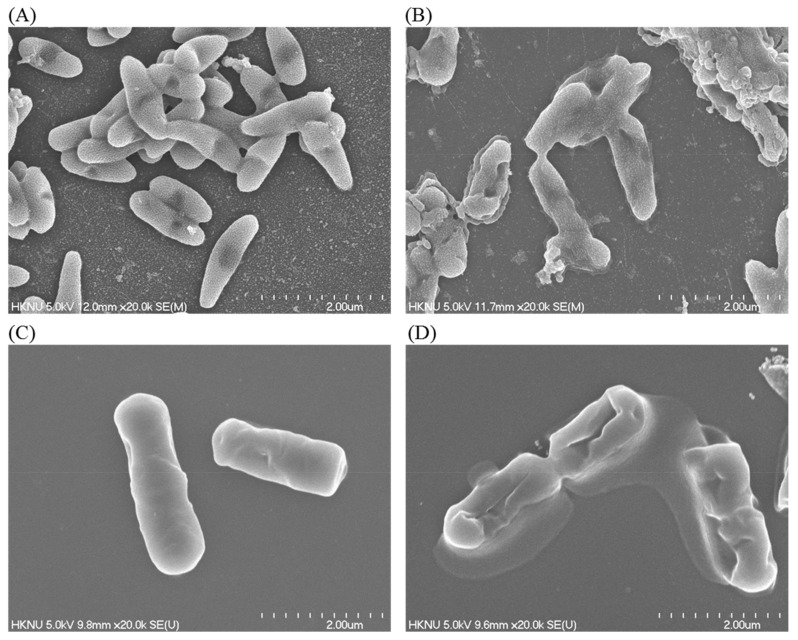
SEM images of normal *P. aeruginosa* cells (**A**), 1 × MBC AgNPs-treated *P. aeruginosa* cells (**B**), normal *B. cereus* cells (**C**), 1 × MBC AgNPs-treated *B. cereus* cells (**D**).

The alterations in the morphology, structure, and surface integrity of *P. aeruginosa* and *B. cereus* cells were investigated by FE-SEM, after treatment with synthesized AgNPs at MBC (25 µg/mL) concentration for 6 h, as shown in Figure 10. The FE-SEM images of normal (untreated) *P. aeruginosa* cells displayed regular rod-shaped with intact cell wall and cell surface without any damage (Figure 10A). However, significant changes were found in the treated cells. AgNPs-treated cells showed damaged, irregular, abnormal, and porous outer surfaces with loss of cell wall integrity (Figure 10B). Similarly, the normal (untreated) *B. cereus* cells displayed regular rod-shaped with intact cell surface without any damage (Figure 10C). On the other hand, the AgNPs-treated cells showed damaged, irregular, abnormal, and porous outer surfaces with loss of cell wall integrity (Figure 10D). The FE-SEM analysis revealed that biosynthesized AgNPs were responsible for damaging the cell wall and cell structure and finally, leading to cell death. The results of current study suggested that the bacteriogenic-synthesized AgNPs may be used to treat both Gram-positive and Gram-negative pathogenic bacteria. Similar FE-SEM observations have been reported earlier as well regarding the effect of biosynthesized AgNPs on pathogenic bacteria [48].

## 4. Conclusions

The bacteria-mediated synthesis of AgNPs is comparatively a non-toxic, ecofriendly, and easy scale-up method. In this study, AgNPs were synthesized by culture supernatant of *P. nicotinovorans* MAHUQ-43 via an extracellular approach to avoid the drawbacks of physico-chemical methods. The FTIR data showed the involvement of different biomolecules during synthesis of AgNPs, as reducing and capping agents. The synthesized AgNPs were crystalline in nature and spherical in shape with the size range of 13–27 nm confirmed by FE-TEM, EDX, and XRD analysis. The synthesis was relatively faster which might be useful for mass production. The *P. nicotinovorans* MAHUQ-43-mediated synthesized AgNPs showed potent antibacterial activity against both *P. aeruginosa* and *B. cereus*. The MIC and MBC for both pathogens were 12.5 µg/mL and 25 µg/mL, respectively. Further, FE-SEM analysis of treated cells showed that biosynthesized AgNPs can damage the cell wall, destroy the membrane integrity and alter the normal morphology of both pathogens, leading to cell death. Thus, *P. nicotinovorans* MAHUQ-43-mediated eco-friendly approach could be used for the rapid, facile, and non-toxic synthesis of AgNPs as an alternative to conventional methods. Moreover, *P. nicotinovorans* MAHUQ-43-mediated synthesized AgNPs showed excellent antibacterial activity against both Gram-positive and Gram-negative bacteria, which suggests their potential application to combat pathogenic microorganisms.

## Figures and Tables

**Table 1:** The number and percentage of chemical elements present in EDX spectrum of *P. nicotinovorans* MAHUQ-43-mediated synthesized AgNPs.

Element	Weight %	Atomic %
Cu K	31.70	44.07
Ag L	68.30	55.93
Totals	100.00	100.00

**Table 2:** Antibacterial efficacy of *P. nicotinovorans* MAHUQ-43-mediated synthesized AgNPs and some commercial antibiotics against *P. aeruginosa* and *B. cereus*.

Pathogenic Species	Zone of Inhibition (mm)				
	AgNO_3_ (30 μL)	AgNO_3_ (60 μL)	Penicillin G	Lincomycin	Vancomycin
*Pseudomonas aeruginosa* [ATCC 10145]	15.5± 0.8	24.7 ± 0.9	-	-	-
*Bacillus cereus* [ATCC 10876]	13.6 ± 0.5	19.3 ± 1.0	-	11.5 ± 0.7	12.2 ± 0.9

## Data Availability

Data are contained within the article and Supplementary Materials.

## Supplementary Materials

The following are available online at https://www.mdpi.com/article/10.3390/ma14102615/s1, Figure S1: Effect of temperature on the biosynthesis of AgNPs using strain *Paenarthrobacter nicotinovorans* MAHUQ-43 was checked on the basis of UV-vis spectral analysis after 24 h of incubation with 1 mM concentration of AgNO_3_; Figure S2: Effect of salt concentration (AgNO_3_) on the biosynthesis of AgNPs using strain *Paenarthrobacter nicotinovorans* MAHUQ-43 was checked on the basis of UV-vis spectral analysis after 24 h of incubation at 30 °C.
