# Supplementary material for: Bacterial Mediated Rapid and Facile Synthesis of Silver Nanoparticles and Their Antimicrobial Efficacy against Pathogenic Microorganisms
Source: Materials (Basel). 2021 May 18;14(10):2615. doi: 10.3390/ma14102615 (PMC8155946; doi:10.3390/ma14102615)
Supplement: Supplementary file 1 [file materials-14-02615-s001.zip › materials-1196959-supplementary.pdf]

materials

MDPI

Supplementary Material

Bacterial Mediated Rapid and Facile Synthesis of Silver Nanoparticles and Their Antimicrobial Efficacy against Pathogenic Microorganisms

Md. Amdadul Huq [1,*] and Shahina Akter [2,*]

[1] Department of Food and Nutrition, College of Biotechnology and Natural Resource, Chung-Ang University, Anseong 17546, Gyeonggi-do, Korea
[2] Department of Food Science and Biotechnology, Gachon University, Seongnam 461-701, Korea
* Correspondence: amdadbge@gmail.com or amdadbge100@cau.ac.kr (M.A.H.); shahinabristy16@gmail.com (S.A); Tel.: +82-031-670-4568 (M.A.H.)

Abstract: In the present study, silver nanoparticles (AgNPs), biosynthesized using culture supernatant of bacterial strain *Paenarthrobacter nicotinovorans* MAHUQ-43, were characterized and their antimicrobial activity was investigated against both Gram-positive *Bacillus cereus* and Gram-negative bacteria *Pseudomonas aeruginosa*. Bacterial-mediated synthesized AgNPs were characterized by UV-Visible (UV-Vis) spectrophotometer, field emission-transmission electron microscopy (FE-TEM), energy dispersive X-ray (EDX), X-ray diffraction (XRD), Fourier transform infrared (FTIR) spectroscopy, and dynamic light scattering (DLS) analysis. The UV-Vis spectral analysis showed the absorption maxima at 466 nm which assured the synthesis of AgNPs. The FE-TEM analysis revealed the spherical shape of nanoparticles with the size range from 13 to 27 nm. The EDX and XRD analysis ensured the crystalline nature of biosynthesized AgNPs. The FTIR analysis revealed the involvement of different biomolecules for the synthesis of AgNPs as reducing and capping agents. The bacterial-mediated synthesized AgNPs inhibited the growth of pathogenic strains *B. cereus* and *P. aeruginosa* and developed a clear zone of inhibition (ZOI). The MIC and MBC for both pathogens were 12.5 μg/mL and 25 μg/mL, respectively. Moreover, field emission scanning electron microscopy analysis revealed that the synthesized AgNPs can destroy the outer membrane and alter the cell morphology of treated pathogens, leading to the death of cells. This study concludes the eco-friendly, facile and rapid synthesis of AgNPs using *P. nicotinovorans* MAHUQ-43 and synthesized AgNPs showed excellent antimicrobial activity against both Gram-positive and Gram-negative pathogens.

Keywords: *Paenarthrobacter nicotinovorans* MAHUQ-43; extracellular synthesis; AgNPs; antimicrobial activity; *Bacillus cereus*; *Pseudomonas aeruginosa*

Citation: Huq, M.A.; Akter, S. Bacterial Mediated Rapid and Facile Synthesis of Silver Nanoparticles and Their Antimicrobial Efficacy against Pathogenic Microorganisms. *Materials* **2021**, *14*, 2615. https://doi.org/10.3390/ma14102615

Academic Editor: Paweł Piotr Pomastowski

Received: 9 April 2021
Accepted: 14 May 2021
Published: 17 May 2021

Publisher's Note: MDPI stays neutral with regard to jurisdictional claims in published maps and institutional affiliations.

Copyright: © 2021 by the authors. Licensee MDPI, Basel, Switzerland. This article is an open access article distributed under the terms and conditions of the Creative Commons Attribution (CC BY) license (http://creativecommons.org/licenses/by/4.0/).

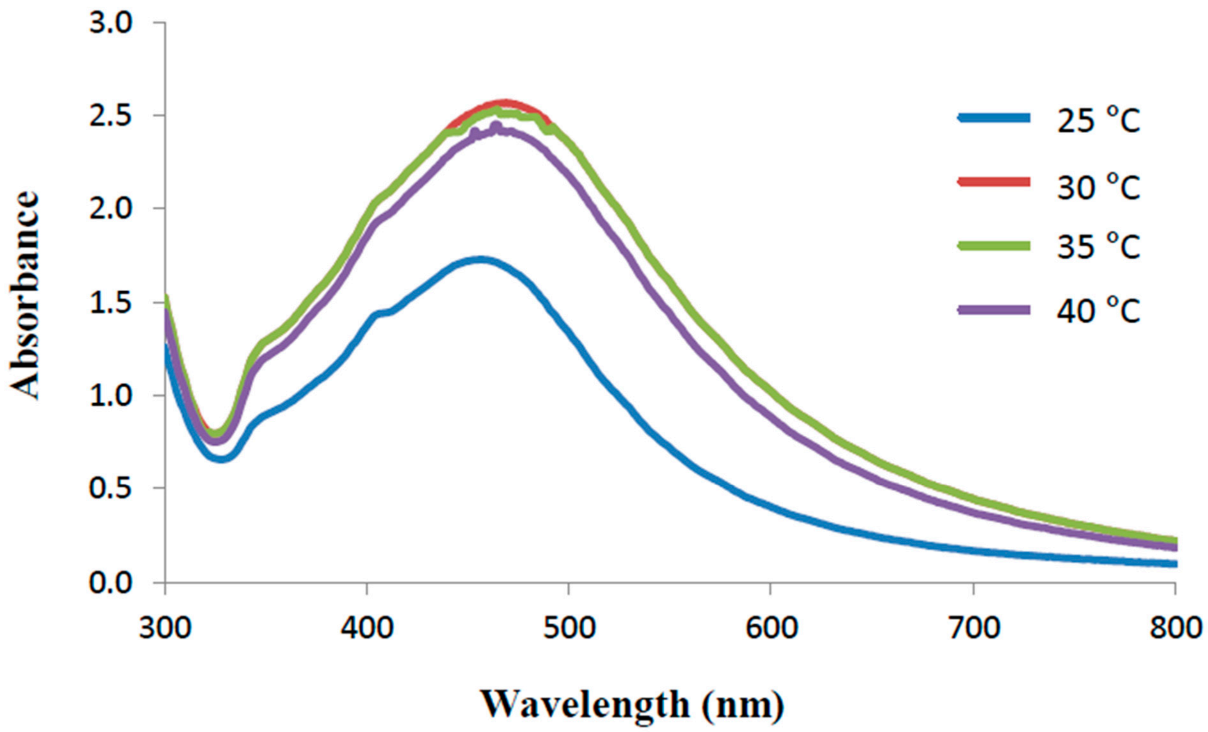

Figure S1. Effect of temperature on the biosynthesis of AgNPs using strain *Paenarthrobacter nicotinovorans* MAHUQ-43 was checked on the basis of UV-vis spectral analysis after 24 h of incubation with 1 mM concentration of $AgNO_3$.

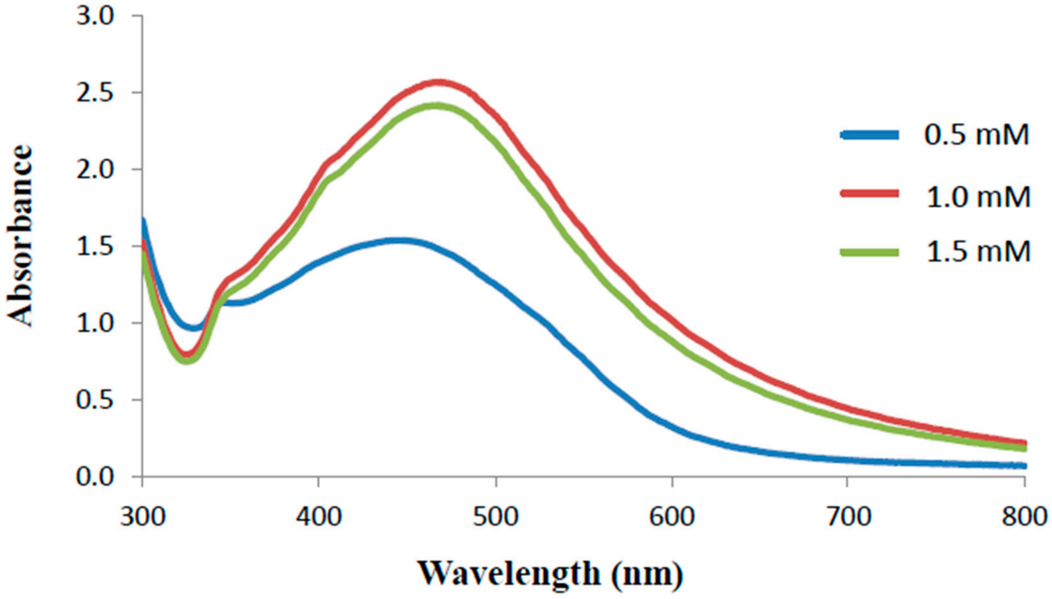

Figure S2. Effect of salt concentration ($AgNO_3$) on the biosynthesis of AgNPs using strain *Paenarthrobacter nicotinovorans* MAHUQ-43 was checked on the basis of UV-vis spectral analysis after 24 h of incubation at 30 °C.
